# Treatment of Compound Fractures

**Published:** 1918

**Authors:** Duncan Eve

**Affiliations:** Nashville, Tenn.


### TREATMENT OF COMPOUND FRACTURES

**By Duncan Eve, A.M., M.D., F.A.C.S., of Nashville, Tenn.**

The last word has not by any means been spoken on the proper treatment of compound fractures.

What is to be done is to some extent determined by the patient's age and general health. If in doubt give the limb (other things considered) the benefit of the doubt and try to save it; should an artery alone be ruptured, cut down upon it and ligate both ends; if a vein alone is torn, suture it, applying a lateral ligature, or even tie both ends; if a nerve is severed, suture same; if a joint is opened, drain and asepticize. Should an effort be made to save the limb, be ready at any moment to amputate for gangrene, secondary hemorrhage, profuse and prolonged suppuration, pyemia, osteomyelitis and necrosis.

If the wound leading to the fracture is small, clean and not contused, as is usually the case in compound fractures by indirect violence, it may with any projecting ends be thoroughly cleansed, and with the skin and tract of wound as far as can be conveniently reached, painted or swabbed with tincture of iodine, the fracture reduced (if possible without enlarging the opening), the wound partially closed by sutures (some not tied at first), and primary union expected. In case the wound heals aseptically the treatment is that of a simple fracture, but if suppuration occurs, the wound must be promptly opened up, thorough drainage provided for at dependent locations, and healing is concluded under management the same as proposed in treatment of granulating wounds.

If, on the other hand, suppuration seems inevitable from the severe contusion and laceration of the soft parts, as well as the dirty condition of the tissues, provision should at once be made for thorough drainage and the part treated with iodine or iodin-guiacol solution; a fluffed, copious sterile gauze dressing applied, and the part placed in a comfortable position of rest, or immobilization without an effort at reduction of the fractured fragments. The guide usually to the investigation and progress of the case, is the temperature. If it remain below 101 degrees F., the wound had best be left untouched for from five to seven days. In the presence of existing suppuration, the most vigorous antiseptic course is indicated, but in doubtful cases the disturbance caused by frequent changes of dressings may induce infection, when it otherwise might not take place.

The necessity of removing detached fragments of bone depends almost entirely upon the prospect of suppuration. In a clean wound fragments that are entirely detached may serve good purpose in the future union of the bone and function of the part. Whereas, in the presence of pus, these small pieces and even the ends of large fragments, may require removal.

Dr. C. H. Blickensderfer (we are glad to state, one of our old pupils), in a recent valuable paper published in the New York Medical Journal, emphasizes as follows: "The successful treatment of compound fractures depends upon three principles: asepsis, drainage, and proper fixation or immobilization. There is more or less destruction and laceration of the soft parts, and more or less extensive fragmentation and comminution of bone. The vascular supply blood-vessels, resulting in a large outpouring of blood and serum. These wounds are therefore highly susceptible to infection. Primary drainage is required, supplemented by efficient counter-drains. The resort to secondary drainage is often imperatively demanded and must be ample because infcetion which is now established, urgently requires the relief by the immediate and complete removal of all infectious matter and secretions resulting from bacterial activity and tissue destruction."

Dr. Chas. H. Mayo, believes it best where possible to attempt to reduce the fracture and convert it as near as possible into a simple one, getting the granulations to proliferate for some days before any special work is done in the way of a bone operation. He claims there is plenty of time to change to the open treatment, anywhere from eight to sixteen days.

Dr. Thomas W. Huntington, suggests the open treatment should be withheld, except where a joint is involved, until the external wound is healed.

Dr. John B. Murphy has, many times and in unmeasured terms, condemned meddlesome interference in compound fractures, and there seems to be a general consensus of opinion, on the part of surgeons of large experience, to the end that it is better to await the permanent closure of the external wound before resorting to radical corrective measures.

Dr. John F. Binnie, in perhaps one of the most recent publications on surgery suggests, "Always remember that the wound is of incomparably greater importance than reduction of the fracture. If reduction of the fracture does not interfere with drainage and wound treatment, then immediate reduction is indicated; if reduction interferes in any way with efficient drainage, then partial reduction plus as thorough as possible immobilization, is the treatment of choice until the drains are removed, when reduction should be carried out. During the after-treatment some form of interrupted (bracketed) splint is a great boon, as it permits the dressings to be changed with the least possible disturbance of the parts.''

Dr. Binnie also suggests the chief aims of immobilization are:

''(1) To maintain reduction.

''(2) To minimize pain and discomfort.

''(3) To facilitate drainage.

''(4) To prevent spread of infection due to movements of muscles and bone fragments, and

'(5) To facilitate the treatment of the infected wound.

''Different methods are therefore applicable in different cases.

If it is thought proper, on account of extraneous matter being forced into the wound at the time of injury, to immediately lay the wound open sufficiently to remove the foreign substance; it must be remembered the part should first be carefully disinfected with iodine, or carbolic acid might be used, the same being neutralized later with absolute alcohol. At this same opportunity loose bone fragments could be removed, however those having attachment to the periosteum should be carefully adjusted b ybeing placed in as nearly a normal position as possible, and if it seems likely that the same can be maintained by the use of external splints alone, of course, employ these supports. If however, it is clear that the position of the fragments cannot be relied upon, then catgut sutures should be employed to fixate the fragments.

After the wound has healed, and there is misplacement or deformity of the fragments, this can be corrected by an open operation, and if the time is late that this open operation is made, it may be possible that an osteotomy will be required. It may also be necessary to use some material for fixation of the fragments. If this is true, let me insist that you avoid the use of metal, especially the once often used Lane plate. We will admit some surgeons use this device yet, and we will have to state with more or less success, but I am glad most surgeons think bone-grafts and some other appliances are much more satisfactory as results prove. Personally, we must confess we are prejudiced, for we cannot forget the hard work undergone and now undergoing, caused by the use of Lane's plates. Understand not from our use in the recent past, for it has been several years since we have employed one. If it is thought that a plate is advisable to be employed in a given case, then use a more delicate and smaller plate, such as Sherman's vanadium spring steel with delicate screws of the same material.

Besides the bone-grafts, there are intramedullary bone plates or tubes, ivory pegs for doweling, ferrules of decalcified bone, and magnesium intramedullary plates or tubes. Then any number of external fixation applicances, such as Freeman's, Lambott's, Hey-Grove's, DePage's and others, that many surgeons advocate.

It is rarely that any fixation appliance is used alone, but nearly always some external support or splint is employed for additional immobilization of the fracture. Among those that are most popular, might be mentioned Binnie's wire "basket-handles," reinforced by plaster-of-paris, and used on both upper and lower extremity. Robert Jones has a very ingeneous splint he has introduced for compound fractures of the humerus. Other good ones for either the upper or lower extremity, are Blake's, Haye's, Lyle's, and Steinman's. One cane easily take the suggestion of Binnie's "basket-handles," or with galvanized iron strips and plaster-of-paris construct what he wants. One of the best splints we have ever employed for a compound fracture of the lower extremity, is Brown's modification of Hodgen's splint, which can be so arranged that it is not only made to satisfy the surgeon, but is most comfortable to the patient.

Dr. John C. A. Gerster, of New York, contributed a very valuable paper on this subject at the last (Atlanta) meeting of this association, which was published in a recent number of the Southern Medical Journal, from which we copy the following:

"The commonly seen compound fracture in which the skin is barely broken, if promptly treated, cleansed, flooded with iodine, reduced and immobilized, usually heals without suppuration.

"An entirely different problem presents itself in the compound fractures filling the military hospitls of Europe. Contrary to expectation, the so-called first-aid measures (iodine and dry sterile gauze), proved of practically no value for preventing suppuration in wounds which were extensively contaminated. The only way to prevent a fatal sepsis was to establish free drainage by a most thorough and radical revision of the wound, so that all gross foreign matter was removed, and every nook and cranny was widely opened. At the first aid stations all they can do is to immobilize the limb (most of the modern armies has excellent skeleton splints for this purpose), and prevent further contamination of the wound, especially when the Carrel-Dakin solution is employed.

"One requisite of the Carrel-Dakin method is that a competent chemist make up the solution fresh every three to seven days. If this is not feasible, an excellent antiseptic solution is that used by Mayer, a surgeon in the French army, and composed of two stock solutions: one containing chlorinated lime 1 part, to 20 parts of a 6% solution of Acetic acid; and the other containing 2 parts of magnesium sulphate, to 20 parts of water. Equal parts are mixed of the two solutions when needed.

"The shortening present in bad compound fractures is best overcome by nail extension, or one of its modifications; and rarely if ever is a plating operation in septic cases done."

Dr. Gester adds a footnote as follows: "Since the reading of this paper it has been the writer's fortune to hear at different time from Drs. H. H. M. Lyle, Charles Powers and George Harley, concerning the Carrel-Dakin sterilization of wounds. There can be no doubt that the few men who actually follow every minute detail of this treatment have been able to obtain the same excellent results as its originators. It is the method of the future in traumatic surgery—in fact, in the treatment of all suppuration.''

Now in conclusion, let us give the technique, or how we would treat in private practice, for example, a compound fracture of the most frequent of all long bones, viz: the tibia.
13—MED___ _____ _____ _____ _____ shrdlu etaoin schmdrlu
In this we will confine our description entirely to the operation. All other details, including an immunzing dose of anti-tetanic toxine, are supposed to have been met.

**Step. 1.** Anesthetize—shave the leg, scrub the whole leg with gasoline or ether to remove grime and grease, then scrub with alcohol, or Harringtons solution. Independent of above, shave off hair (dry) and paint with tincture of iodine. Apply an elastic constrictor.

**Step 2.** Enlarge the skin wound freely so that the deep wound becomes accessible. With gauze sponges, gloved finger, and instruments, remove all goreing material, blood clots, etc. Dissect away the ragged skin around the wound also all portions of tissues so injured that they cannot live. May have to remove loose peices of bone. Fragments attached by periosteum should be cleansed and retained. Remove all tissue which will interfere with union by being interposed between the frgments. With retractors open all torn tissue planes and spaces. If much dirt has gained access, mop out the whole wound with pure carbolic acid and immediately follow this by mopping with pure grain alcohol. Instead of using the carbolic acid you can mop out with 50 per cent. tincture of iodine. Provide free drainage for dependent parts of the wound and make counter-punctiures if necessary. Reduce the fracture. If required fixate with catgut or a bonegraft. Remove the elastic constrictor. Attend to hemostasis by means of forceps, ligature and hot, sterile water. See that drainage tubes are in place and not clogged.

**Step. 3.** Close the external wound. Dress and immobilize.—Southern Practitioner.

